# The impact of interactions between heavy metals and smoking exposures on the formation of oral microbial communities

**DOI:** 10.3389/fmicb.2024.1502812

**Published:** 2024-12-18

**Authors:** Qiwen Zheng, Yonghua Zhang, Jia Li, Shuwei Pei, Jiangyun Liu, Lu Feng, Li Zhang, Xingrong Liu, Bin Luo, Ye Ruan, Weigang Hu, Jingping Niu, Tian Tian

**Affiliations:** ^1^School of Public Health, Lanzhou University, Lanzhou, Gansu, China; ^2^Child Health Department, Lanzhou Maternal and Child Health Care Hospital, Lanzhou, Gansu, China; ^3^School of Stomatology, Lanzhou University, Lanzhou, Gansu, China; ^4^State Key Laboratory of Herbage Improvement and Grassland Agro-Ecosystems, College of Ecology, Lanzhou University, Lanzhou, China

**Keywords:** bacteria, buccal mucosa, heavy metals, smoking, interactions

## Abstract

**Introduction:**

The primary objective of our investigation was to assess the repercussions of prolonged exposure to heavy metals and smoking on the microbiome of the oral buccal mucosa. Concurrently, we aimed to elucidate the intricate interplay between external environmental exposures and the composition of the oral microbial ecosystem, thereby discerning its potential implications for human health.

**Methods:**

Our study cohort was stratified into four distinct groups: MS (characterized by concurrent exposure to heavy metals and smoking), M (exposed solely to heavy metals), S (exposed solely to smoking), and C (comprising individuals serving as a control group). Specimens of buccal mucosa and blood were systematically acquired from the participants, facilitating subsequent microbial diversity analysis across the four oral buccal mucosa sample cohorts through 16S rRNA gene sequencing techniques. Simultaneously, blood samples were tested for heavy metal concentrations. In addition, we performed topological analyses by constructing microbial networks.

**Results:**

Our findings notably indicate that co-exposure to heavy metals and smoking yielded a more pronounced alteration in the diversity of oral microflora when compared to singular exposures to either heavy metals or smoking. By comparing the oral bacterial communities and functional pathways between the four groups, we found significant differences in bacterial communities and functional pathways between the groups. Notably, the impact of heavy metal exposure overshadowed that of smoking, with concurrent exposure to heavy metals and smoking eliciting marginally greater effects than exposure to heavy metals alone. In addition, our analysis of the correlation between microbiota and blood heavy metal concentrations showed that the heavy metal cadmium (Cd) had a significantly greater effect on oral microbiota than other heavy metals.

**Discussion:**

Chronic exposure to heavy metals and smoking disrupts the normal bacterial communities in the oral mucosa of residents of contaminated areas. This exposure reduces the complexity and stability of microbial networks and increases the risk of various diseases reduces the complexity and stability.

## 1 Introduction

Oral health is an important part of systemic health. As an important first gateway to the human body, the oral cavity not only undertakes complex functions such as chewing, swallowing, and speech, but also has a close connection with a number of systems such as the respiratory system, digestive system, and cardiovascular system (Sedghi et al., 2021; Zhang et al., 2018). At the same time, the oral cavity is one of the most diverse sites in the human body for hosting microorganisms and is one of the five research priorities of the Human Microbiome Project (HMP) (oral, nasal, vaginal, intestinal, and skin) (Li X. et al., 2022). The oral microbiome plays a critical role in health and disease. Heavy metals are important raw materials that contribute to the development of national economies. However, the environmental impact of heavy metal emissions from metal production processes is of increasing concern worldwide. In particular, areas around mining and smelting facilities are often heavily polluted by heavy metals, causing irreversible ecological damage (Li K. et al., 2022). Heavy metals that have been discharged accumulate in soil and water and eventually enter the human body through the food chain or through direct contact with the skin; excessive accumulation of these metals in the human body can have carcinogenic effects, affect vital organs and ultimately disrupt normal biological functions (Li et al., 2024). With the rapid development of industrialization, urbanization and agricultural intensification, heavy metal contamination of soil has become an important global environmental problem and has attracted much attention (Liu et al., 2025). Although small amounts of copper and zinc are essential elements, they can be toxic to humans and animals at high concentrations. Lead, cadmium, mercury and arsenic are non-essential elements that may be mutagenic, teratogenic and carcinogenic at very low levels (Bi et al., 2018). Mercury (Hg), Arsenic (As), Lead (Pb), Cadmium (Cd), and Chromium (Cr) have drawn great attention owing to their poisoning risks and high toxicity (Sha et al., 2019). Cd is known for its harmful effects on kidneys and bones, while As is highly toxic to most multicellular organisms (Zhou et al., 2024). In addition, recent studies have shown that heavy metal pollution also affects the structure of human microbial communities.

The oral cavity contains a variety of microbial communities, including bacteria, archaea, fungi and viruses, which are closely related to the occurrence of oral diseases (Pietrobon et al., 2021). Under normal physiological conditions, the oral flora maintains a dynamic balance, which can resist the invasion of exogenous pathogenic bacteria and protect the health of the organism (Li B.J. et al., 2023; Hoashi-Takiguchi et al., 2022). When the balance of oral flora is broken under the action of certain stimulating factors, the relative abundance of pathogenic bacteria in the oral cavity increases and the relative abundance of beneficial bacteria decreases (Liu et al., 2021), and the microenvironment of the oral cavity changes (Priya et al., 2020), which leads to periodontal disease (Hathaway-Schrader and Novince, 2021), caries (Zhang et al., 2018) and other oral diseases. Studies have validated that dysbiosis of the oral flora not only precipitates oral diseases but also exhibits a close correlation with the onset and progression of diverse systemic diseases, encompassing cardiovascular ailments such as atherosclerosis and myocardial infarction (Wolf and Ley, 2019; Marietta et al., 2019), respiratory diseases (Tada and Senpuku, 2021) such as chronic obstructive pulmonary disease (COPD) and lung cancer, neurological diseases (Lin et al., 2021) such as Alzheimer’s disease, digestive diseases (Gao et al., 2018) such as inflammatory bowel disease (IBD) and gastrointestinal tract cancer (GI), endocrine diseases (Ruff et al., 2020) such as diabetes mellitus and polycystic ovary syndrome (PCOS), and immune system diseases such as HIV.

In general, oral microorganisms in healthy individuals are in a state of equilibrium with temporal stability. The oral bacterial community is dominated by six phyla: Firmicutes, Bacteroidota, Proteobacteria, Actinobacteriota, Fusobacteriota and Spirochaetes (Dewhirst et al., 2010). While microorganisms establish a stable ecological niche in the human host, they are at the same time susceptible to a variety of internal and external factors, such as antibiotics, dietary habits, smoking and exposure to heavy metals. Firstly, antibiotics not only significantly affect the composition and function of the oral microbiota, but also induce specific metabolic changes during antibiotic interventions (Monroy-Perez et al., 2020). A prospective cohort study found that the Shannon diversity index was reduced after treatment with amoxicillin relative to the untreated group and continued to be reduced over 6 months (Menon et al., 2019). Secondly, numerous scholars have endeavored to discern the correlation between diet and oral microbiota in the pursuit of formulating dietary regimens conducive to oral health. In infants, oral candidiasis is less frequent in breastfed and mixed-fed infants than in those fed solid foods: in adults, dietary changes can also modulate the oral microbiota and prevent the development of associated diseases (Anderson et al., 2020). Thirdly, smoking is widely recognized to affect the composition of the oral microbiota. For instance, smokers exhibit a notable reduction in the abundance of Proteobacteria within the oral microbiota, coupled with an elevation in Firmicutes and Actinobacteriota (Wu et al., 2016). Fourthly, epidemiological evidence indicates a correlation between exposure to toxic metals and oral microbiome-mediated diseases, such as dental caries and gingival diseases. Moreover, toxic metals such as antimony, arsenic, and mercury in saliva have been linked to alterations in the composition of the oral microbiome. Specifically, elevated levels of antimony and increased quantities of Lactobacillus spp. have been associated with dental caries. Heavy metals (Cr, Ni, Cu) are associated with the growth of *Capnocytophaga*, *Neisseria*, *Aggregatella*, *Streptococcus*, *Campylobacter*, *Selenomonas* and *Prevotella* in the oral cavity, and the heavy metals induce changes in the structure of the bacterial community (Zhang W. et al., 2023).

These factors mentioned above affect oral microbial communities to different degrees. A total of 127 participants were screened to collect oral samples based on the inclusion criteria, our objectives are to (1) determine which factors affect the microbial community structure of the human oral cavity; (2) explore the differences in the composition and diversity of oral microbial communities under the influence of heavy metal contamination and cigarette smoking; (3) predict functional differences in oral bacterial communities; and (4) assess whether network topological features of oral microbial communities differ under heavy metal and smoking exposure.

## 2 Materials and methods

### 2.1 Research site

Long-term smelting and mining of non-ferrous metals in the last century have seriously polluted the atmosphere, soil, surface water and groundwater in Baiyin City. Minqin and Shuanghe villages (36°28′38.188″N, 104°18′47.870″E; 36°27′24.650″N, 104°21′22.057″E) are selected as typical polluted areas. As a comparison, Hewan and Yangwa villages (35°46′41.541″N, 104°0′37.443″E; 35°45′54.661″N, 104°1′28.117″E), which are located 100 km away from Baiyin City and have relatively low levels of heavy metal pollution, in Yuzhong County, Lanzhou City, were selected as control areas. These two selected areas have similar levels of socio-economic development, and their inhabitants have similar lifestyles and eating habits.

### 2.2 Collection of soil samples and heavy metal analysis

**Figure 1:**
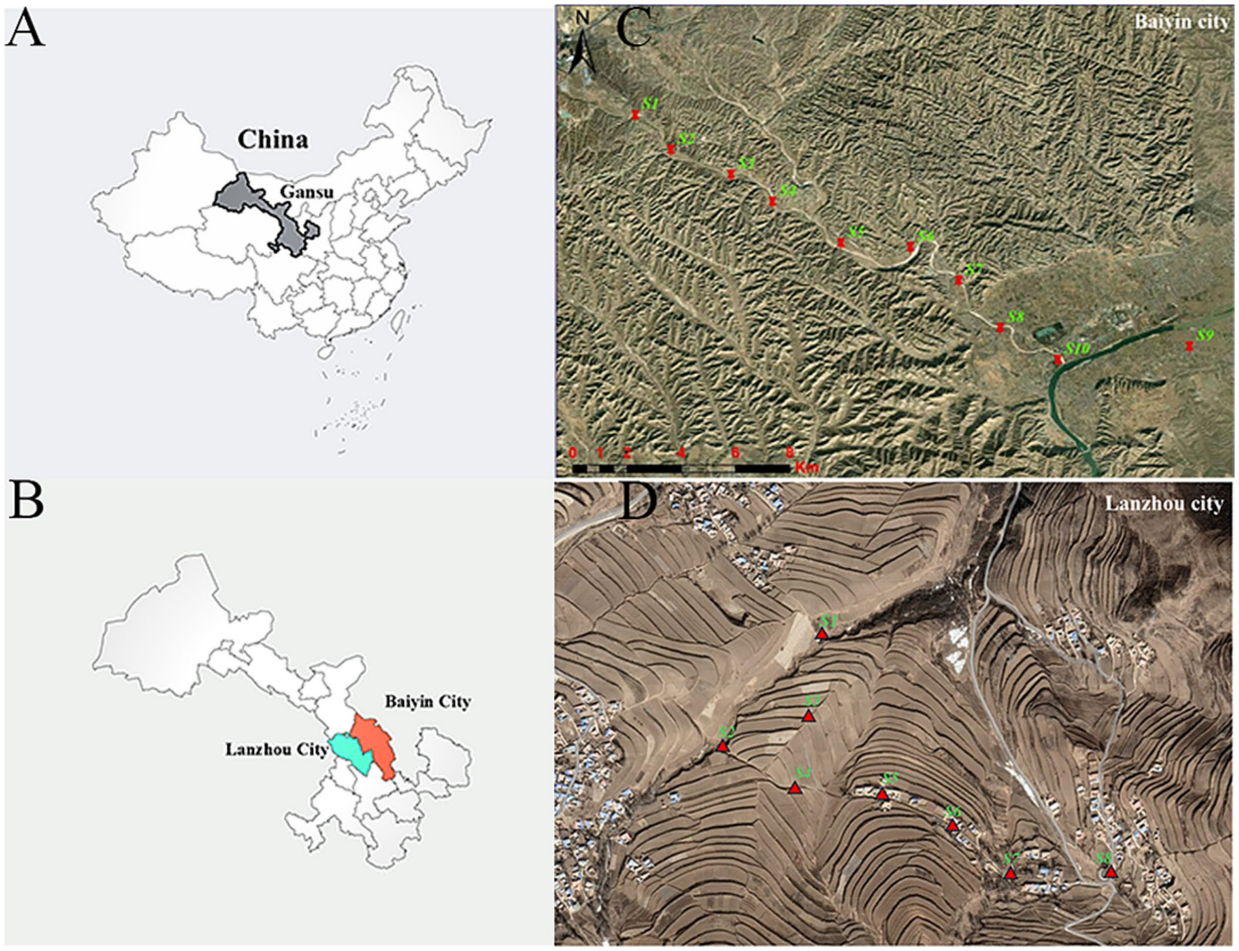
The location of sampling points in the contaminated and control areas. **(A)** China, **(B)** Baiyin and Lanzhou are shown on the left. The geographical locations of the **(C)** contaminated and **(D)** control sampling sites are shown on the right.

Soil samples were collected in April 2021 from the contaminated and control areas to assess the levels of heavy metal pollution. A total of 18 sampling points were selected in this study (S1–S10, S1–S8), with S1–S10 located in the field in the vicinity of Minqin village and Shuanghe village (Figure 1C) and S1–S8 located in the field near Yangwa village and Hewan village (Figure 1D). At each sampling point, areas of approximately 10 × 10 m were randomly selected in the fields, and five subsampling sites were set up in each selected field using a five-point sampling method. After removing gravel and impurities at the surface, soil from five subsampling points (at 20 cm depths) was collected using a sterile wooden spatula and thoroughly mixed into a composite sample. A total of 18 samples were collected. The samples were sent to the laboratory on the same day. To determine the heavy metal content in soil, each soil sample was first air-dried at room temperature, and then biological debris, plant roots, leaves, and stones were removed, followed by sieving through a 200-mesh nylon sieve. Finally, each sample was thoroughly mixed and stored in a polyethylene bag for further analysis. Each sample of approximately 0.5 g was digested using a microwave digestion system (Sartorius, PB-10, Germany). Then, the content of heavy metals (Mn, Sb, Cu, Cd, Zn, Hg, Pb, Mo, Co, and Ni) was measured using inductively coupled plasma–mass spectrometry (ICP–MS, Agilent, United States). Quality assurance/control procedures were conducted using standard reference materials (Chinese Academy of Measurement Science) with each batch of samples (one blank and one standard).

### 2.3 Research object

During the period from September 2019 to January 2021, residents who have lived in the local area for more than 10 years, who have not left the local area for more than half a consecutive year to go out to work, etc., and who were aged 40–69 years old, who voluntarily participated in the present survey and signed the informed consent form, were included by questionnaire in the two regions, and comprehensive demographic and lifestyle information was collected using questionnaire surveys, and the relevant information about the researchers’ targets was determined. The questionnaire covered three main sections: lifestyle habits, smoking behavior and oral health. By excluding those with missing information from the questionnaire or insufficient biological samples, a total of 127 subjects were included and divided into four groups: MS = (co-exposure to heavy metals and smoking) = 34, M = (exposure to heavy metals) = 55, S = (exposure to smoking) = 19, and C = (control group) = 19.

### 2.4 The collection of oral buccal mucosa and blood samples

Fasting venous blood was collected from all subjects in vacuum anticoagulated blood collection tubes. The blood samples were gently mixed upside down to prevent coagulation. Subsequently, 1 mL of whole blood was divided into 2 mL freezing tubes for the analysis of heavy metal content, and the separated blood samples were stored at −80°C for measurement.

The oral samples were collected from the study subjects meeting the following four criteria: (1) no systemic diseases such as diabetes mellitus, hypertension, etc.; (2) no history of medication (including antibiotics) or basic periodontal treatment in the 3 months prior to the collection of oral samples; (3) the number of teeth in the mouth was ≥24; and (4) no oral diseases, such as dental caries, periodontal disease, oral cancer, etc. Smokers need to have smoked for at least 10 years from the legal smoking age. Subjects were informed in advance not to eat for at least 1 h before sampling and were allowed to rinse their mouths with water to remove food debris in the mouth before sampling. Then, the buccal mucosa on the left and right sides of their mouths were scraped with a sterile cotton swab for 10 s. After oral sampling, all samples were immediately stored at −80°C for subsequent laboratory processing.

To investigate the association between the concentration of heavy metals in the blood and the composition of the buccal mucosa microbiota, blood samples were collected from the peripheral veins of the research subjects. We collected 15 mL of heparinized venous blood, removed 2 mL of whole blood, and stored it at −80°C. The contents of heavy metals in blood were measured using an inductively coupled-mass spectrometer (ICP–MS, Elan DRC-II ICP–MS, PerkinElmer Sciex, United States). Subjects signed informed consent and the study was approved by the Ethics Committee of Lanzhou University School of Public Health.

### 2.5 DNA extraction, sequencing and bioinformatic analyses

DNA was extracted from each buccal mucosa sample using an E.Z.N.A. Soil DNA Kit (Omega Bio-Tek, Norcross, GA, United States) following the manufacturer’s instructions, and its concentration and purity were assessed on a 1% agarose gel. The hypervariable region V3–V4 of the bacterial 16S rRNA gene were amplified with primer pairs 338F (5′-ACTCCTACGGGAGGCAGCAG-3′) and 806R (5′-GGACTACHVGGGTWTCTAAT-3′) by an ABI GeneAmp® 9700 PCR thermocycler (ABI, CA, United States). Thermocycling conditions consisted of 3 min at 95°C, followed by 30 amplification cycles of 30 s denaturation at 95°C, 30 s annealing at 55°C, 72°C for 45 s, and a final extension of 72°C for 10 min. All amplification reactions were performed in a total volume of 20 μL, containing 4 μL of 5× FastPfu Buffer, 2 μL of 2.5 mM dNTPs, 0.8 μL of both the forward and reverse primers, 10 ng of template DNA, and 0.4 μL of FastPfu DNA Polymerase. To mitigate individual PCR biases, each sample was amplified in triplicate and pooled together. The amplicon quality of the PCR products was assessed on a 2% agarose gel, followed by purification with an AxyPrep Gel Extraction Kit (Axygen Biosciences, United States). Purified amplicons were combined at equimolar concentrations and paired-end sequenced (2 × 300 bp) on an Illumina MiSeq platform (Illumina, United States) at the Majorbio Bio-pharm Technology Co., Ltd. (Shanghai, China) according to standard protocols. Raw sequencing data of the bacterial 16S rRNA gene have been deposited in the NCBI Sequence Read Archive under BioProject accession number PRJNA979792. The resulting sequences were processed using the QIIME pipeline. Briefly, low-quality sequences were trimmed with Cutadapt and quality filtered. Paired-end reads were assembled using FLASH version 1.2.11. USEARCH was used to remove chimeric sequences based on the UCHIME algorithm, and the remaining sequences were allocated to operational taxonomic units (OTUs) with 97% similarity using the UPARSE pipeline. OTUs with fewer than two sequences were eliminated, and their representative sequences were assigned to taxonomic lineages using the RDP classifier version 2.2 against the SILVA database (version 138) using confidence threshold of 0.7.

### 2.6 Statistical analysis

Alpha diversity index ACE, Shannon index, etc. were calculated using Qiime software. The Wilcoxon rank sum test was employed for intergroup differences in Alpha diversity. The similarity of microbial community structure among samples was examined using PCoA analysis (Principal Coordinate Analysis) based on the Bray–Curtis distance algorithm. This analysis was combined with the PERMANOVA non-parametric test to determine whether the differences in microbial community structure between sample groups were significant; LEfSe analysis (Linear discriminant analysis Effect Size) (LDA > 3.5, *p* < 0.05) was used to identify bacterial taxa with significant differences in abundance from phylum to genus level between different groups. To assess functional differences in metabolic pathways in microbial communities between groups, we used PICRUSt2 based on the SILVA database of 16S rRNA sequences and the Kyoto Encyclopedia of Genes and Genomes (KEGG) database to predict microbial functional genes. Given the potential for a non-linear response relationship between mixture exposures, BKMR was used to assess the combined effects of different exposure factors on the bacterial and KEGG pathways. This approach integrates Bayesian and statistical learning methods to estimate nonlinearities and/or interactions in exposure-outcome associations. The analyses were conducted using the bkmr package in R Statistical Software. A total of 50,000 iterations of the BKMR variable selection model were performed using the Markov chain Monte Carlo algorithm. Response relationships for individual exposures and mixed exposure effects were investigated using BKMR, Including exploring bidirectional interactions between the main exposures.

### 2.7 Network analysis

Ecological network analysis was able to reveal the co-occurrence patterns between different microorganisms. To eliminate rare OTUs, those with a mean relative abundance less than 0.01% across all samples were removed. Spearman rank correlation were calculated between pairs of OTUs, and *p*-values from the correlation analysis were adjusted using the Benjamin and Hochberg False Discovery Rate (FDR) controlling methods. The meta-community network was constructed using the weighted correlation network analysis (WGCNA) package, based on the correlation coefficients and FDR-adjusted *p*-values. A cutoff of 0.001 for *p*-values (FDR-adjusted) and a threshold of 0.77 for correlation coefficients were selected using the methods dependent on random matrix theory. Each node in the network represents one OTU, and each edge that connects two nodes represents the correlation between OTUs. Network topological features were obtained with the “igraph” package. All the samples were then divided into four groups. Sub-network images of each group were visualized using the Gephi 0.9.2.^1^ To characterize the network topology, we calculated four node-level topological features (i.e., closeness centrality, node degree, betweenness centrality, and eigen centrality) and six network-level topological features (i.e., nodes, links, cluster number, average degree, graph density, and modularity) for each sub-network.

## 3 Results

### 3.1 The heavy metal pollution of the study area

To assess whether there were differences in metal pollution levels between the contaminated and control areas, the concentrations of heavy metals in the soil and the blood of the subjects in the two areas were compared using the Wilcoxon rank-sum test. In the ploughed soil of the contaminated area, our results showed that the mean values of seven metals (Mo, Cd, Sb, Cu, Hg, Pb, and Zn) were substantially higher than those of the control area (*p* < 0.05), whereas the levels of Co, Ni, and Mn were similar between the two areas (*p* > 0.05; Supplementary Table S1). The concentrations of four metals (Zn, Hg, Cd and Pb) in the blood of subjects living in contaminated areas were significantly higher than those in the control area (*p* < 0.05; Supplementary Table S2).

### 3.2 Factors affecting the oral microbiota

Following the removal of low-quality sequences, chloroplasts, chimeras, and singleton sequences, a total of 3,602,609 high-quality reads were obtained from 127 human oral samples, with an average of 28,367 reads per sample. Within the scope of the present investigation, a comprehensive tally of 2,300 Operational Taxonomic Units (OTUs) was discerned and classified into 39 phyla, 108 classes, 260 orders, 439 families, and 901 genera. Analysis via PERMANOVA revealed a significant influence of heavy metal exposure and smoking on the community structure and composition of oral bacteria (*p* < 0.05), whereas dyspepsia and antibiotic usage did not yield a significant effect on the community structure and composition of bacteria (*p* > 0.05; Supplementary Table S3). Consequently, our study pivoted toward investigating the impacts of heavy metals and smoking on oral microorganisms.

### 3.3 Alpha and beta diversity of the oral microbiota

#### 3.3.1 Rarefaction curve

**Figure 2:**
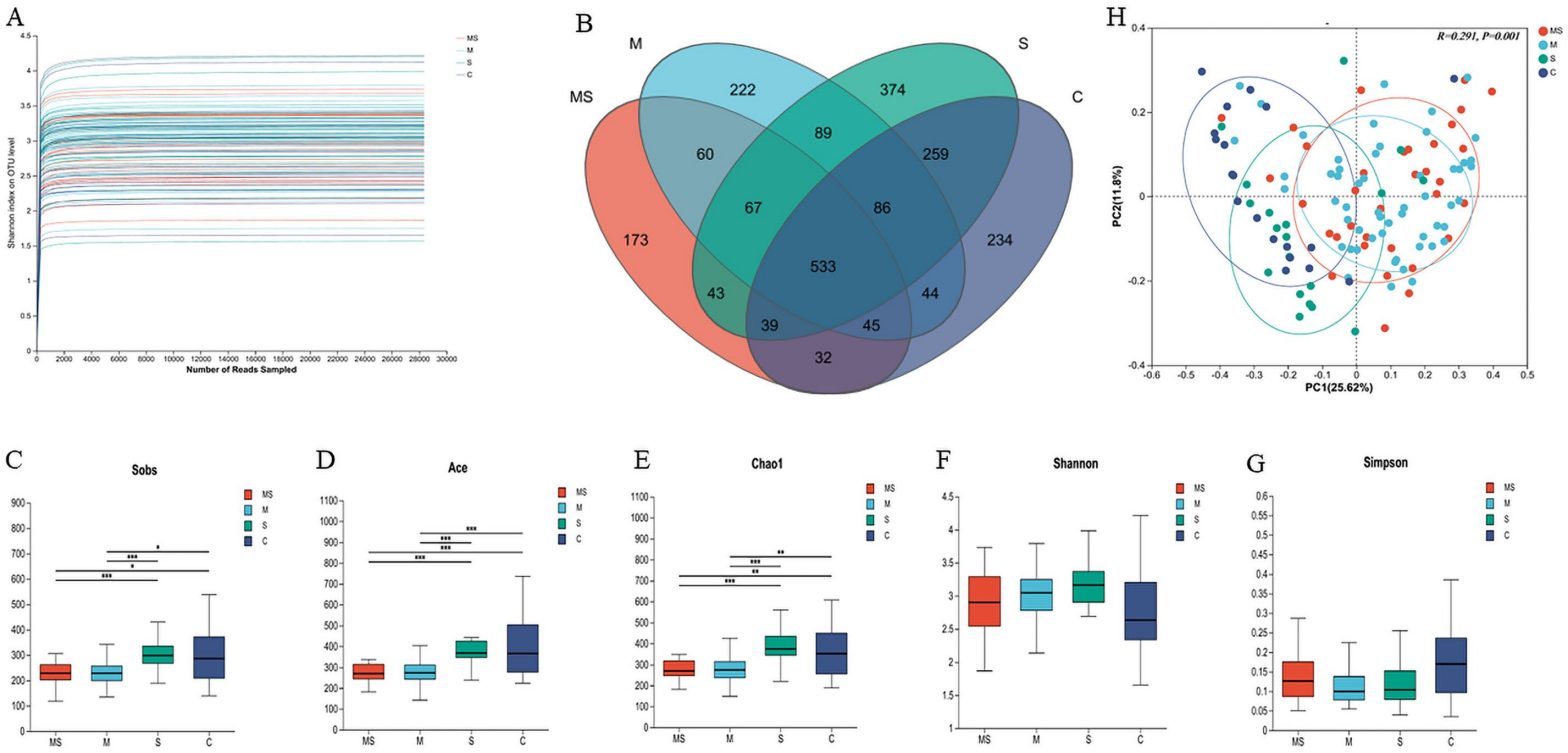
Analysis of α-diversity and principal coordinate analysis of buccal mucosal bacterial communities. **(A)** Rarefaction curves for the bacterial community dataset. The flat curve indicated that the sequence number used for analyses was adequate. **(B)** Number of common and unique OTUs in oral samples. **(C–G)** Differences in bacterial α diversity indices between the MS, M, S, and C groups. Significant differences were determined by using the Wilcoxon rank-sum test. *p ≤ 0.05; **p ≤ 0.01; ***p ≤ 0.001. **(H)** Principal coordinate analysis (PCoA) of bacterial community dissimilarities based on the Bray–Curtis distances. Significant differences in bacterial β diversity between the MS, M, S, and C groups were determined by using ANOSIM statistics (MS = co-exposure to heavy metals and smoking, M = exposure to heavy metals, S = exposure to smoking, and C = control group).

The rarefaction curves were plotted to evaluate whether the sequencing volume was sufficient to cover all taxa, indirectly reflecting the richness of species in the samples and the depth of sequencing. By analyzing the dilution curves of the samples (Figure 2A), it could be seen that the curves of each group are relatively flat, which indicated that the data obtained from sequencing were well saturated to meet the requirements of the analysis, and that the sequencing data could reflect the diversity information of oral bacterial communities well.

#### 3.3.2 Venn diagram

The Venn diagram could visualize the similarity and overlap status of the composition of the number of OTUs in the oral samples. The numbers of shared and unique OTUs in oral samples under different exposure conditions were shown below (Figure 2B). It was found that the number of shared OTUs among different groups in oral samples was *N* = 533, which accounting for 23.17% of the total number of OTUs. Moreover, the highest number of shared OTUs was found in group S and control (*N* = 917, 39.87%), while the lowest number of shared OTUs was found in group MS and control (*N* = 649, 28.22%). This suggested that the effect of mixed exposure to heavy metals and smoking on the number of OTUs in oral samples was less than the effect of exposure alone.

#### 3.3.3 Alpha diversity of oral bacterial communities

For the Alpha diversity of human oral microbial communities, ACE is an index used to estimate the number of microorganisms in the environment (the species richness of the community), and the larger its value, the higher the microbial richness in the community; Shannon is an index used to estimate the species diversity of the environmental community, and the larger its value, the higher the diversity of the community. We found that the ACE index was the largest in group C, indicating the highest microbial richness in this group, and that the ACE index differed significantly among the four groups (*p* < 0.01). However, the Shannon and Simpson indices showed no statistically significant difference (Figures 2C–G).

#### 3.3.4 Beta diversity of oral bacterial communities

Based on the Bray–Curtis distance matrix, the microbial community structure of the different groups in the oral samples was as follows (Figure 2H and Supplementary Table S4). The differences in microbial structure between the different groups in the oral samples were significant (*R*^2^ = 0.2906, *p* = 0.001). Specifically, there was a significant difference in microbial structure between any two groups in the oral samples (*p* < 0.05). The largest difference in microbial communities was found between the MS and control groups (*R*^2^ = 0.205, *p* = 0.001), while the smallest difference in communities was found between the S and control groups (*R*^2^ = 0.070, *p* = 0.01). The differences in microbial communities gradually increased in the order of group S, M and MS compared to the control group.

### 3.4 Bacterial community structure of oral samples

A total of 2,300 OTUs were identified in this study, belonging to 39 phylums, 108 classes, 260 orders, 439 families and 901 genera.

**Figure 3:**
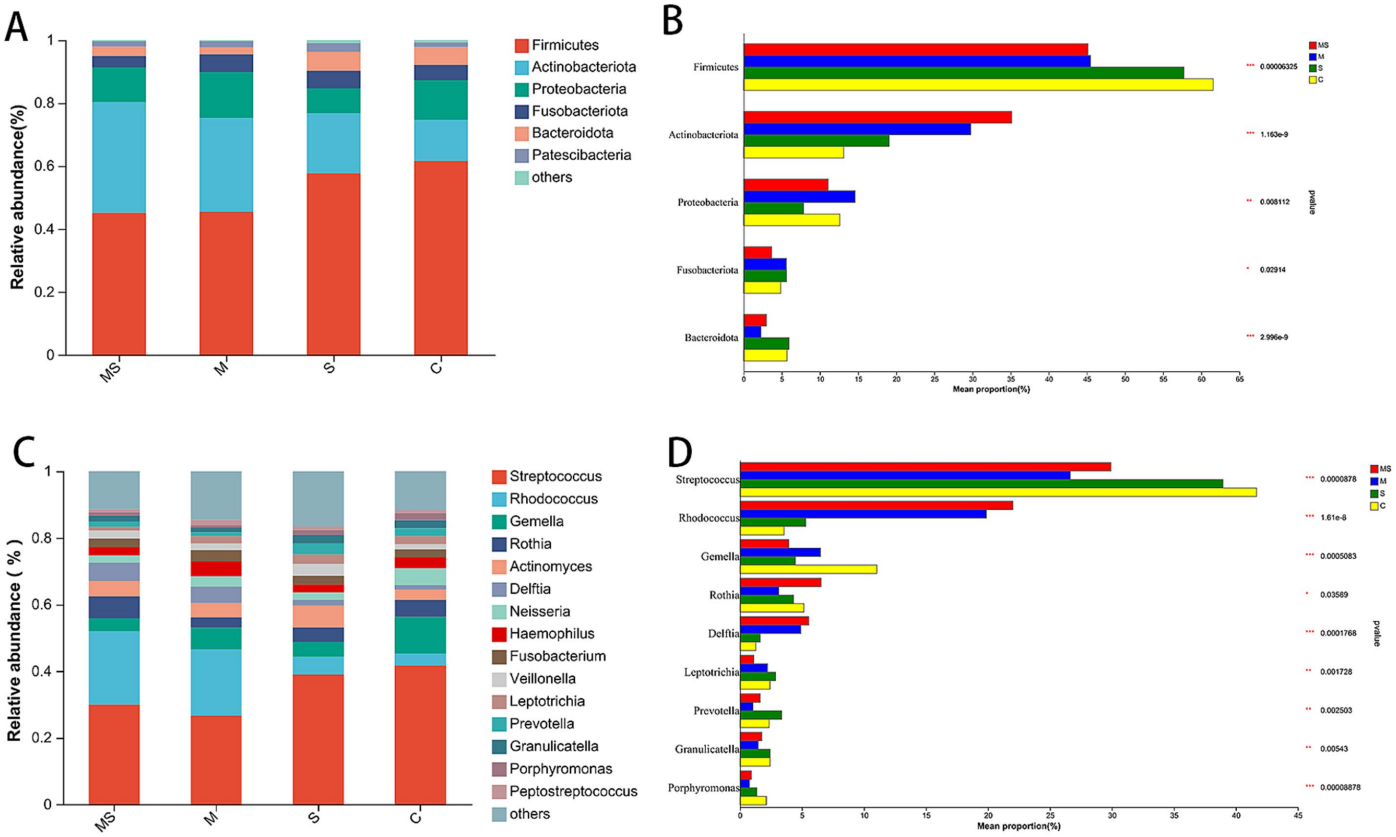
The bacterial community composition of the MS, M, S, and C groups. Compositional differences in bacterial communities of the buccal mucosa at the phylum **(A,B)** and genus **(C,D)** levels. The *p*-value was calculated using the Wilcoxon rank-sum test and adjusted by using the false discovery rate. *p < 0.05; **p < 0.01; ***p < 0.001 (MS = co-exposure to heavy metals and smoking, M = exposure to heavy metals, S = exposure to smoking, and C = control group).

At the phylum level, a total of 39 phyla were detected, and the phyla with relative abundance >1% were Firmicutes, Actinobacteriota, Proteobacteria, Fusobacteriota, Bacteroidota, Patescibacteria. The first 5 of these phyla accounted for more than 95% of the oral flora of all samples and were the main dominant phyla in the human oral flora (Figures 3A,B and Supplementary Table S5). The abundance of Firmicutes gradually increased in the order of group MS, M, S, and C; while the abundance of Actinobacteriota gradually decreased in the order of group MS, M, S, and C.

At the genus level, a total of 901 genera were detected, and the genera with relative abundance >1% were *Streptococcus*, *Rhodococcus*, *Gemella*, *Rothia*, *Actinomyces*, *Delftia*, *Neisseria*, *Haemophilus*, *Fusobacterium*, *Veillonella*, *Leptotrichia*, *Prevotella*, *Granulicatella*, *Porphyromonas*, *Peptostreptococcus* (Figures 3C,D and Supplementary Table S6). Among them, the relative abundance of *Streptococcus* in group C was higher than that in all other groups; the abundance of *Rhodococcus* gradually decreased in the order of group MS, M, S, and C.

### 3.5 Analysis of differential species

**Figure 4:**
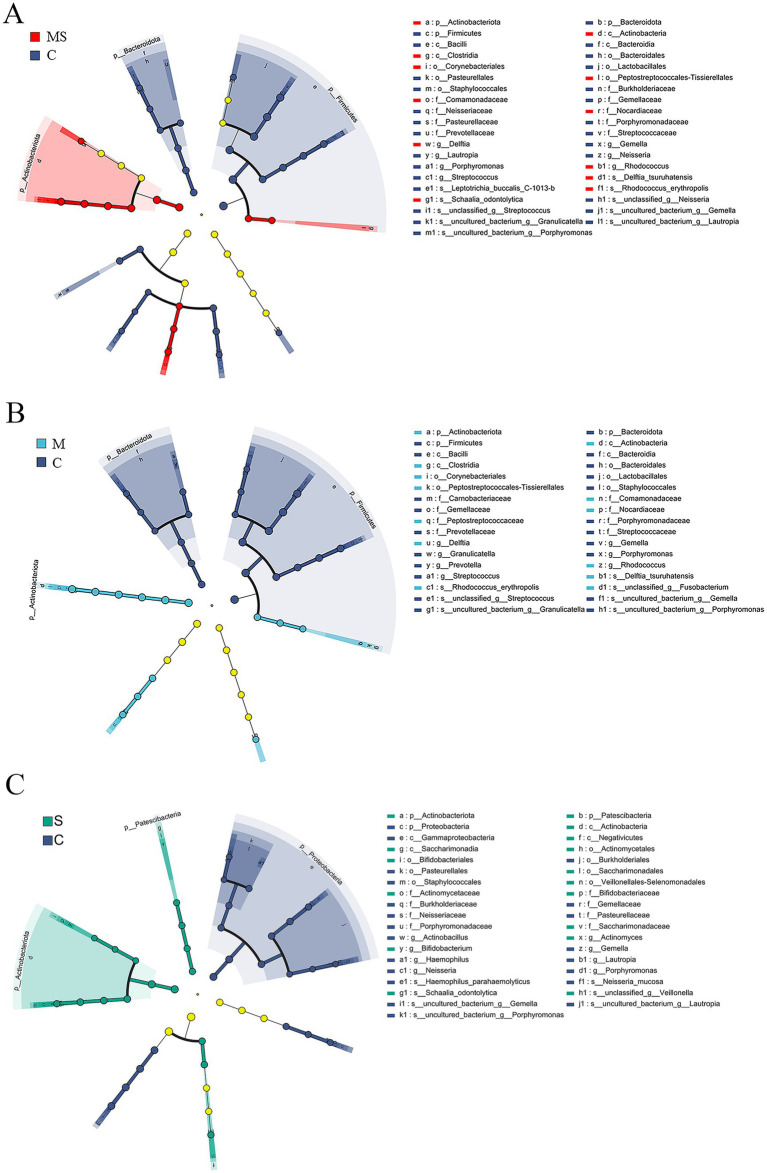
LEfSe analysis to select species that differed between groups. **(A)** Branch diagram of lefse difference species between MS and C groups. **(B)** Branch diagram of lefse difference species between M and C groups. **(C)** Branch diagram of lefse difference species between S and C groups (MS = co-exposure to heavy metals and smoking, M = exposure to heavy metals, S = exposure to smoking, and C = control group).

The samples were analyzed for intergroup differences using LEfSe analysis to look for species that marked significant differences at each taxonomic level. Differences in the dominant phylum and genus of microbial communities between groups were as follows (Figure 4; Supplementary Figure S1; Table 1).

**Table 1:** Differences in relative abundance of dominant phyla and genera of microbial communities between groups (*p < 0.05, **p < 0.01, and ***p < 0.001).

ANOVA	Phyla	*p*	Genera	*p*
			Gemella	0.001**
	Actinobacteriota	0.023*	Actinomyces	0.020*
C vs. S	Proteobacteria	0.036*	Neisseria	0.017*
	Patescibacteria	0.034*	Haemophilus	0.041*
			Porphyromonas	0.021*
			Streptococcus	<0.001***
			Rhodococcus	<0.001***
	Firmicutes	<0.001***	Gemella	0.002**
C vs. M	Actinobacteriota	<0.001***	Delftia	<0.001***
	Bacteroidota	<0.001***	Prevotella	<0.001***
			Granulicatella	0.003**
			Porphyromonas	<0.001***
			Streptococcus	0.004**
	Firmicutes	<0.001***	Rhodococcus	<0.001***
	Actinobacteriota	<0.001***	Gemella	<0.001***
C vs. MS	Bacteroidota	<0.001***	Delftia	<0.001***
			Neisseria	0.004**
			Porphyromonas	<0.001***

At the phylum level, after LEfSe analysis, three dominant phyla were found to be significantly different between groups C and S: Actinobacteriota, Proteobacteria, and Patescibacteria; and three dominant phyla found to be significantly different between groups C and M: Firmicutes, Actinobacteriota, and Bacteroidota. The differential species found between groups C and MS were the same as those found in groups C and M. The relative abundance of Actinobacteriota was significantly (*p* < 0.05) higher in groups S, M and MS compared to Group C. In addition, the relative abundance of Firmicutes and Bacteroidota was significantly lower (*p* < 0.001) in both groups MS and M. The relative abundance of *Proteobacteria* was significantly lower (*p* < 0.05) in group S than in group C, whereas that of Patescibacteria was significantly higher (*p* < 0.05).

At the genus level, five dominant genera were found to be significantly different between Groups C and S: *Gemella*, *Actinomyces*, *Neisseria*, *Haemophilus*, and *Porphyromonas*; seven dominant genera were found to be significantly different between Groups C and M: *Streptococcus*, *Rhodococcus*, *Gemella*, *Delftia*, *Prevotella*, *Granulicatella*, and *Porphyromonas*; significant differences were found between groups C and MS for six dominant genera: *Streptococcus*, *Rhodococcus*, *Gemella*, *Delftia*, *Neisseria*, and *Porphyromonas*. The relative abundance of *Gemella* and *Porphyromonas* was significantly reduced in groups S, M and MS compared to group C (*p* < 0.05); the relative abundance of *Rhodococcus* and *Delftia* was significantly increased in groups M and MS (*p* < 0.001). In addition, the relative abundance of *Neisseria* and *Haemophilus* was significantly (*p* < 0.05) reduced in group S compared to group C; the relative abundance of *Streptococcus*, *Prevotella*, and *Granulicatella* was significantly (*p* < 0.05) reduced in group M; the relative abundance of *Streptococcus* and *Neisseria* were significantly reduced in relative abundance in group MS (*p* = 0.004).

### 3.6 The predictive function analysis using KEGG

**Figure 5:**
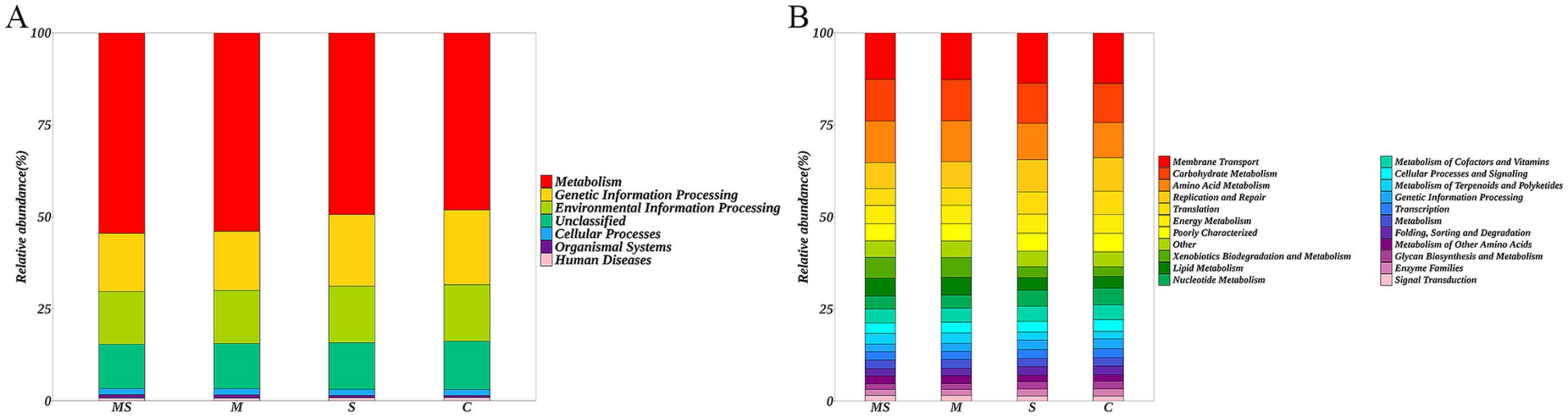
Histogram of the distribution of KEGG level 1 functional pathway genes **(A)** and level 2 functional pathway genes **(B)** among different groups (MS = co-exposure to heavy metals and smoking, M = exposure to heavy metals, S = exposure to smoking, and C = control group).

The differences in microbial gene functions among the four groups in terms of metabolic pathways were assessed using KEGG enrichment analyses. The KEGG database is a comprehensive database that correlates genetic and functional information, and the KEGG PATHWAY database, which analyses metabolic pathways, is one of its core databases. Comparing the non-redundant gene set with the KEGG database, this study annotated the genes to the KEGG database and found that the level 1 KEGG pathways mainly involved seven functional pathways, among which the highest abundance pathway was Metabolism, with an average relative abundance of annotated genes of 51.53% (Figure 5A). Among the 41 level 2 KEGG pathways, Membrane Transport was the pathway with the highest abundance of annotated genes, and the relative abundance of annotated genes was 13.23%, in addition, Carbohydrate Metabolism, Amino Acid Metabolism, Replication and Repair, Translation, and Energy Metabolism had higher average relative abundance of annotated genes of 11.02, 10.44, 7.99, 5.40, and 5.06%, respectively (Figure 5B).

**Figure 6:**
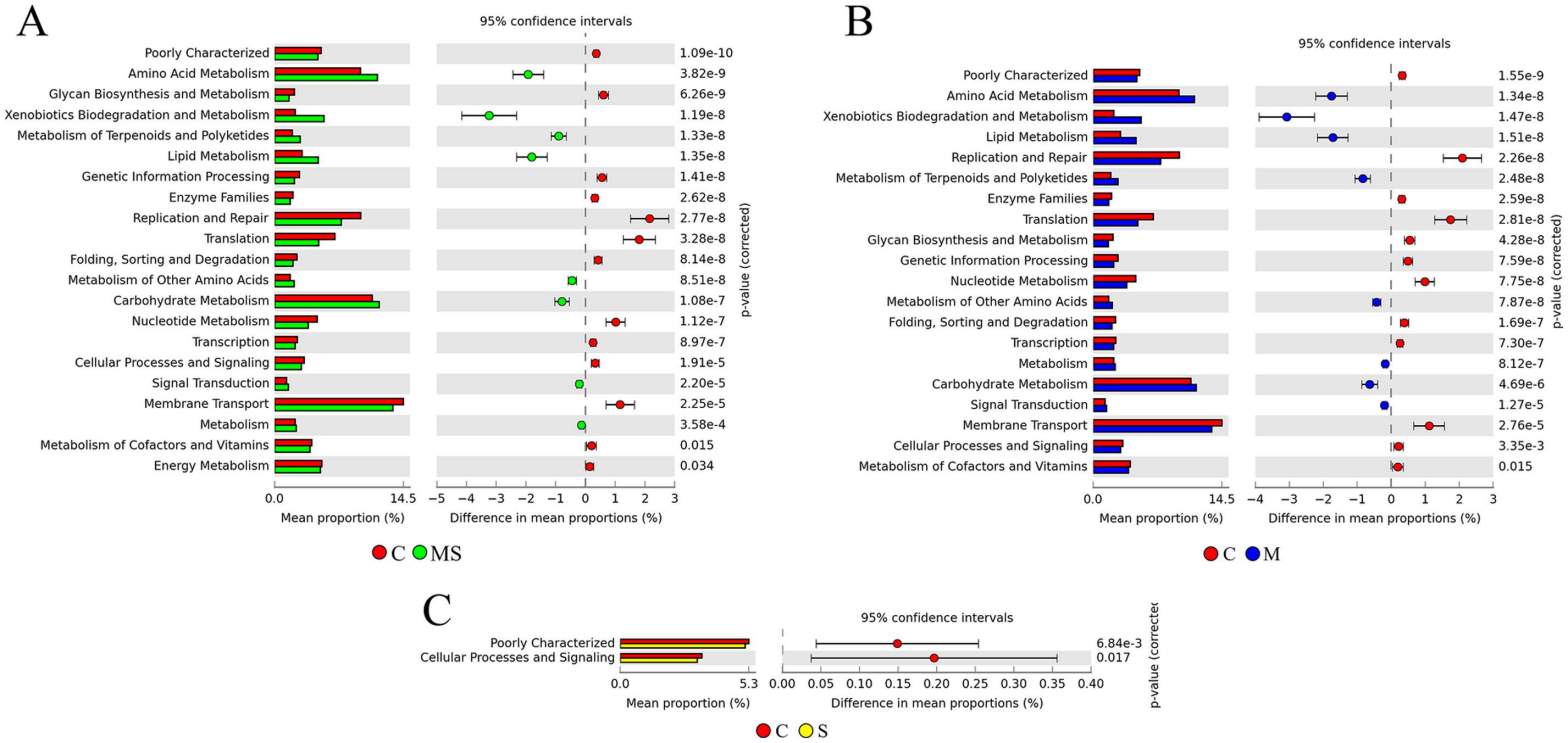
The KEGG pathway predicts differences in microbial community metabolic pathways between groups. **(A)** Differences in predicted function based on KEGG metabolic pathway at level 2 between MS and C groups. **(B)** Differences in predicted function based on KEGG metabolic pathway at level 2 between M and C groups. **(C)** Differences in predicted function based on KEGG metabolic pathway at level 2 between S and C groups (MS = co-exposure to heavy metals and smoking, M = exposure to heavy metals, S = exposure to smoking, and C = control group).

Based on the KEGG level 2 pathways, there was a significant difference between the MS, M and S groups compared to the C group (Figure 6). Comparison of enriched KEGG pathways between the C and S groups indicated that cellular processes and signaling, poorly characterized pathways were enriched in the C group. Comparison of enriched KEGG pathways between the C and S groups indicated that cellular processes and signaling, poorly characterized pathways were enriched in the C group. Further comparison revealed that replication and repair, translation, membrane transport and nucleotide metabolism pathways were enriched in the C group relative to the MS group, while xenobiotics biodegradation and metabolism, amino acid metabolism, lipid metabolism and carbohydrate metabolism were enriched in the MS group than the C group. At the same time, we found similar results in the MS and M groups.

### 3.7 Correlation analysis between oral microbiota and blood heavy metals

**Figure 7:**
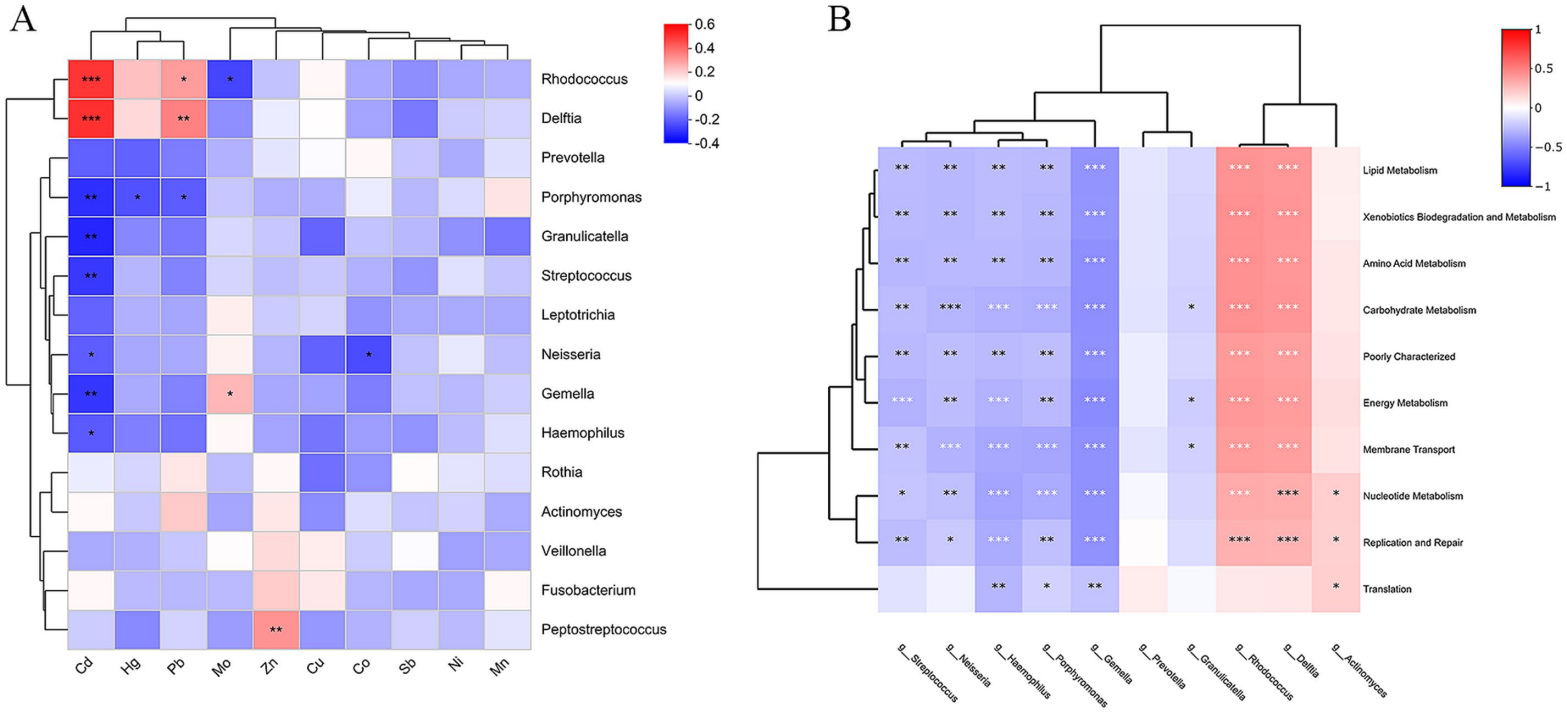
**(A)** Correlation between genus level bacteria and heavy metals. **(B)** Heatmap for correlation analysis of differential bacteria with differential functional pathways (*p < 0.05, **p < 0.01, ***p < 0.001; *p*-values calculated using the SparCC algorithm).

To further investigate the effects of heavy metal exposure on oral microorganisms, Spearman correlation analyses were performed between the measured heavy metal concentrations and genus-level relative abundance of Top 15 bacteria (Figure 7A). The results showed that 6 heavy metals (Cd, Hg, Pb, Mo, Zn, and Co) had a significant effect on the bacterial community. *Rhodococcus* and *Delftia* were positively correlated with Cd and Pb, *Rhodococcus* was negatively correlated with Mo; *Porphyromonas* was negatively correlated with Cd, Hg, and Pb; *Granulicatella*, *Streptococcus*, and *Haemophilus* were negatively correlated with Cd; *Neisseria* was negatively correlated with Cd and Co; *Gemella* was negatively correlated with Cd and positively correlated with Mo; and *Peptostreptococcus* was positively correlated with Zn.

### 3.8 Correlation analysis of differential bacteria with differential functional pathways

In order to clarify the relationship between differential bacteria and differential functions, 10 differential marker species at the genus level were correlated with the top10 differential functional pathways in gene abundance (Figure 7B). The results showed that *Rhodococcus* (belonging to the Actinobacteriota), *Delftia* (belonging to Proteobacteria) was associated with Membrane Transport, Carbohydrate Metabolism, Amino Acid Metabolism, Replication and Repair, Energy Metabolism, Xenobiotics Biodegradation and Metabolism, Lipid Metabolism, Nucleotide Metabolism pathways had strong positive correlations (*p* < 0.001); *Actinomyces* (belonging to the Actinobacteriota) had strong positive correlations with Nucleotide Metabolism, Replication and Repair, Translation pathways were positively correlated (*p* < 0.05). *Streptococcus*, *Gemella*, *Peptostreptococcus*, *Porphyromonas*, *Haemophilus*, and *Neisseria* were strongly negatively correlated with these 10 functional pathways (*p* < 0.05); *Granulicatella* was negatively correlated with Carbohydrate Metabolism, Energy Metabolism, and Membrane Transport (*p* < 0.05).

### 3.9 Ecological network analysis of oral flora

**Figure 8:**
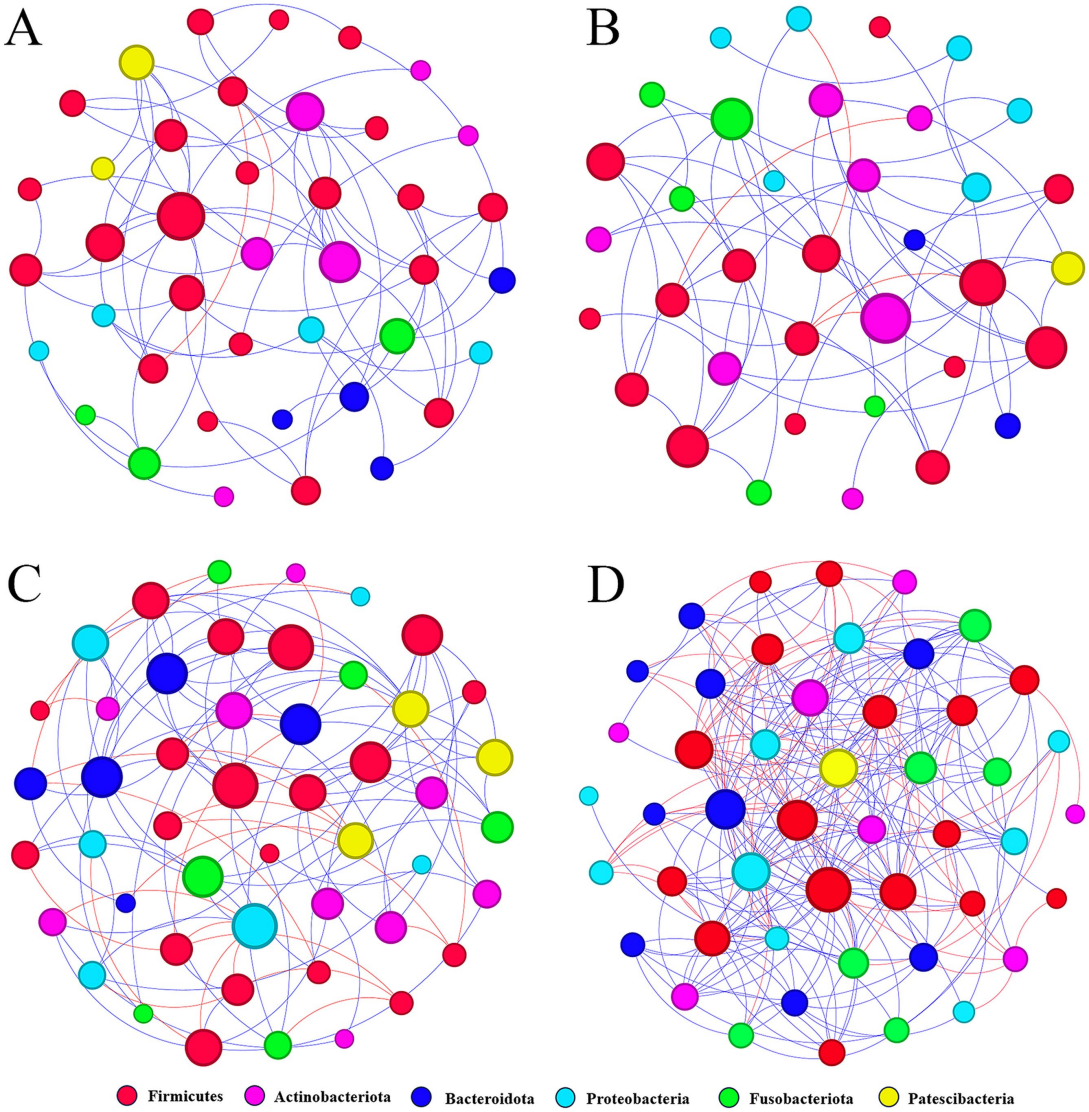
Molecular ecological networks were constructed based on the correlation between bacterial OTUs. The networks of MS **(A)**, M **(B)**, S **(C)**, and C **(D)** are shown. Node sizes are proportional to the number of connections. Each node represents a bacterial OTU and is colored according to its phylum-level classification relationship. Red lines indicate negative interactions between bacterial OTUs, while blue lines indicate positive interactions (MS = co-exposure to heavy metals and smoking, M = exposure to heavy metals, S = exposure to smoking, and C = control group).

A meta-community ecological network was constructed based on the Spearman correlation and then divided into four different sub-networks. These networks consisted of four groups, namely, heavy metal and smoking co-exposure (Figure 8A), heavy metal exposure (Figure 8B), smoking exposure (Figure 8C), and control (Figure 8D). The overall network level characteristics of the microbiota sub-networks were compared between the different exposure groups (Table 2). Compared with the C group, the number of network nodes and edges decreased in the order of group S, group MS, and group M, indicating that the network pattern of group S was more complex and stable; the clustering coefficient of group MS was the highest; the average degree and graph density of group S were the largest; and the modularity and average path length of group M were higher than those of other groups, which indicated that the oral bacterial network of group M was stronger and better connected than those of other groups.

**Table 2:** Network-level topological features of the bacterial subnetworks in human oral.

Group	Nodes	Edges	Clustering coefficient	Average degree	Graph density	Modularity	Average path length
MS	41	72	0.505	3.515	0.088	0.6	3.189
M	36	56	0.39	3.111	0.089	0.628	4.068
S	48	113	0.38	4.708	0.1	0.546	3.134
C	48	249	0.582	10.375	0.221	0.284	2.068

MS = co-exposure to heavy metals and smoking, M = exposure to heavy metals, S = exposure to smoking, and C = control group.

In oral bacteria of group MS, 72 correlations were detected with 70 positive and two negative correlations; in oral bacteria of group M, 56 correlations were detected with 52 positive and four negative correlations; in oral bacteria of group S, 113 correlations were detected with 89 positive and 24 negative correlations; in oral bacteria of group C, 249 correlations with 182 positive and 67 negative correlations. In all four groups, the most positive correlations were found in the *Firmicutes* (*p* < 0.05), and the most negative correlations were found between the *Firmicutes* and the *Actinobacteriota* (*p* < 0.05).

### 3.10 Assessment of exposure effects and interactions of different exposure factors using the BKMR model

**Figure 9:**
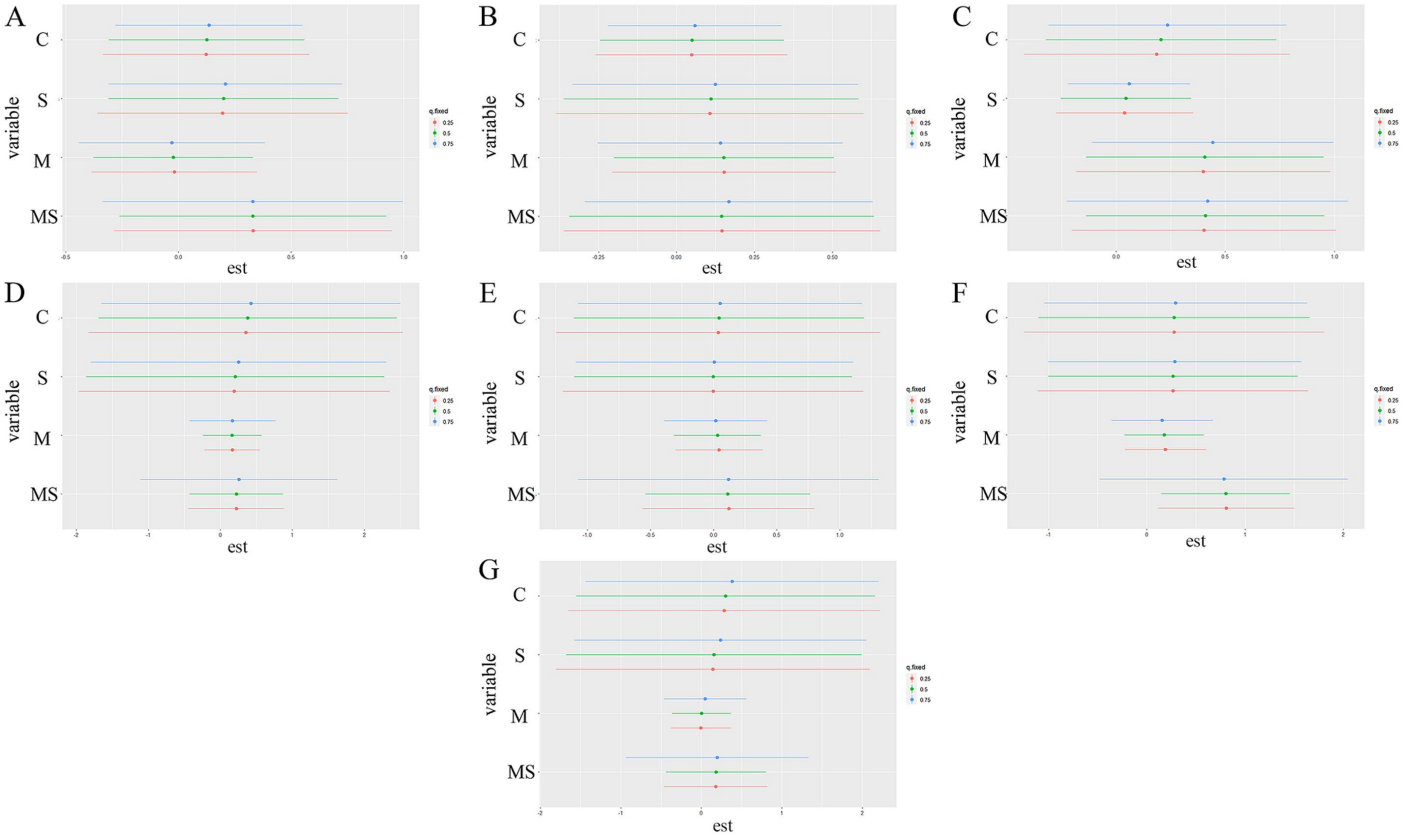
Univariate exposure-response functions for each exposure factor on bacteria phylum levels and KEGG primary pathways. The results were assessed by Bayesian Kernel Machine Regression (BKMR) models. The first three graphs show the effect on the phylum level of bacteria, these are the following Actinobacteriota **(A)**, Patescibacteria **(B)**, and Proteobacteria **(C)**. The latter are based on the effects on the KEGG pathway, respectively, namely Cellular Processes **(D)**, Environmental Information Processing **(E)**, Metabolism **(F)**, and Human Diseases **(G)**.

A BKMR model was used to explore the effects of combined heavy metal and smoking exposure group (MS), heavy metal exposure group (M), smoking group (S), and control group (C) on the abundance of bacteria and KEGG metabolic pathways. In the bacterial phylum classification, the combined heavy metal and smoking exposure group (MS) had a greater effect on *Actinobacteriota* (Figure 9A), *Patescibacteria* (Figure 9B) and *Proteobacteria* (Figure 9C) than the heavy metal exposure group (M); in the KEGG metabolic pathway, the combined heavy metal and smoking exposure group (MS) had a greater effect on the Cellular Processes (Figure 9D), Environmental Information Processing (Figure 9E), Metabolism (Figure 9F) and Human Diseases (Figure 9G) than the heavy metal exposure group (M). Using bivariate-interaction plots, we found no interaction between the different exposure factors (Supplementary Figure S2).

## 4 Discussion

Modulated by factors such as the immune system, host lifestyle, hygiene practices, and environmental conditions, the human oral microbiota had been intricately linked to a diverse spectrum of diseases (Qian et al., 2019; Hurley et al., 2019; Sanz-Martin et al., 2017). Hence, comprehending the oral microbiota holds paramount significance in elucidating the intricate interplay between microbiota and human health. PERMANOVA analysis revealed that heavy metal exposure and smoking had a significant effect on the community structure and composition of oral bacteria, whereas age, gender, indigestion, and antibiotic use did not have a significant effect on the community structure and composition of bacteria. Previous investigations into the oral microbiota have predominantly examined heavy metals and smoking as distinct exposure factors, with limited exploration into their combined effects. Therefore, our study endeavored to delineate the impacts of exposure to heavy metals and smoking individually, as well as in combination, on the constitution of the oral microbiome.

In general, ecosystems with higher species diversity are more stable, functional and self-regulating (Xun et al., 2019; Pennekamp et al., 2018). Although species interacted differently in different ecosystems, there was evidence that high species diversity could provide more functional redundancy and buffer ecosystem functions against possible species loss or extinction when faced with environmental disturbances (Bannar-Martin et al., 2018; Louca et al., 2018). Within the oral microbial system, various oral microbiota were presumed to offer a distinct repertoire of enzymes, facilitating the degradation of toxic substances and constraining the proliferation of specific pathogenic bacteria linked to oral diseases through competitive interactions. Conversely, a decline in oral microbial diversity had been correlated with the occurrence of oral diseases (Zheng et al., 2020). In our study, we observed that the control group exhibited the highest level of species richness. This observation suggested that exposure to heavy metals and cigarette smoke might introduce toxicants, thereby impeding bacterial growth and consequent species depletion. Conversely, simultaneous exposure to heavy metals and cigarette smoke was associated with the highest level of species diversity. This outcome implied that metal exposure may engender an upsurge in metal resistance genes, thereby influencing the composition of bacterial profiles. In addition, cigarette smoke altered the composition of the bacterial flora. The abundance of Neisseria was decreased by smoke (Jia et al., 2021; Wu et al., 2016), which is consistent with our results. Exposure factors contributed to alterations in the oral environment, thereby fostering a diverse array of bacterial habitats. The combined impact of the two factors might precipitate an augmentation in alpha diversity within the oral microbial community. Comparative analyses of beta diversity values among oral microbial communities exposed to different factors revealed a gradual escalation in structural disparities relative to control groups, progressing in the sequence of smoking-exposed, heavy-metal-exposed, and mixed-exposed cohorts. Exposure to heavy metals and smoking might elicit distinct physiological and immune responses in individuals, potentially exacerbating inter-individual variations in oral microbiota. This suggests that heavy metals and smoking affect the diversity of oral bacteria. Metal exposure had a greater impact on microbial diversity than smoking. This might be due to the fact that metals can be highly toxic even in small concentrations. They directly interfered with microbial metabolism, enzymes, and cellular structure, often causing in cellular damage, stress responses, or even death. Many microbes required metals as cofactors for various enzymes. Obligate human pathogens and commensals had to generally derive these from their hosts. Unlike smoking-related chemicals, many metals did not degrade easily. Metals could persist in the environment, and their concentration could accumulate over time, causing chronic exposure to microbial communities. This constant exposure to metals could lead to selective pressure, promoting certain resistant microbial species while eliminating others, reducing overall microbial diversity.

Microbial community profiling revealed that the predominant microorganisms in the oral communities of smokers tended to be Actinobacteriota, Fusobacteriota, Bacteroidota, and Patescibacteria compared to controls. This finding was consistent with a previous study (Yu et al., 2019). According to previous reports (Lim et al., 2018), oral diseases could usually be predicted by the composition and characteristics of microbial communities in oral buccal mucosa samples. Previous studies had shown that smoking disrupts the balance of oral commensal microbial composition, leading to gingival disease and dental alterations (Galvin et al., 2023; Wang et al., 2022). These factors assumed a pivotal role in restructuring the oral microbial community, underscoring the key role of smoking on the homeostasis of microbial communities within the oral cavity. Studies had revealed that individuals exposed to heavy metals exhibit a prevalence of Actinobacteriota, Proteobacteria, Fusobacteriota and Patescibacteria as dominant microorganisms within oral communities, aligning with findings reported in previous research (Zhang W. et al., 2023). The beneficial outcomes stemming from the symbiotic relationship between host and microbiota might bolster host defenses and uphold gastrointestinal tract health. However, microenvironmental shifts triggered by the infiltration of heavy metals into the oral cavity have the potential to disrupt the advantageous effects of host-microbiota symbiosis (Kilian et al., 2016; Rodriguez-Rabassa et al., 2018). When present in relatively high abundance in the corresponding oral communities, Actinobacteriota phylum was commonly associated with intestinal diseases and had been linked to cardiovascular diseases, chronic obstructive pulmonary disease (COPD), asthma, and metabolic disorders (diabetes) (Rizzatti et al., 2017; Larsen et al., 2015). This also implied that a high abundance of Actinobacteriota in the heavy metal and smoking co-exposure group (MS) might disrupt the homeostasis of the oral microbial community, thereby predisposing individuals to oral diseases or infections. At the genus level, the microbial composition of the smoking group exhibited an increase in Actinobacteriota and Patescibacteria, alongside a decrease in Proteobacteria. Similarly, the microbial composition of the heavy metal-exposed group demonstrated an increase in Rhodococcus and Delftia, coupled with a decrease in Streptococcus, Gemella, Prevotella, Granulicatella, and Porphyromonas. Research had elucidated that arsenic exposure correlates with a notable reduction in species within the genus Prevotella, while mercury exposure was associated with significant reductions in species within the genera Neisseria, Granulicatella, and Abotrophia, alongside significant increased in Streptococcus. Furthermore, in a murine model, zinc deficiency and arsenic exposure individually induced alterations in the gut microbiome, and their combined effect exhibited synergistic actions (Gaulke et al., 2018). This might be relevant because the oral and gut microbiomes were predictive of each other (Segata et al., 2012). A previous study (Gao et al., 2018) demonstrated that *Streptococcus* (Firmicutes), *Actinomyces* (Actinobacteria), and portion of *Neisseria* (Proteobacteria) are considered to be normal flora in a healthy oral cavity and can establish a co-operative relationship with the host. We needed to make a special note that although many Neisseria species are commensals in the human microbiota, particularly in the oral and nasopharyngeal regions, it was important to note that certain species, such as meningitidis and gonorrhoeae, are obligate human pathogens and can cause serious infections. Additionally, the presence of Corynebacterium genus had been associated with a reduced risk of laryngeal cancer (Hayes et al., 2018), but this genus was not found in any of the exposed groups. The genus *Porphyromonas* (belonging to the Bacteroidetes) was thought to be most often positively associated with the etiology of periodontitis and other oral diseases (Costalonga and Herzberg, 2014).

The composition of oral salivary flora exhibited variations among different groups, necessitating a thorough investigation into the functional gene diversity of oral flora to comprehend the impact of changes in flora composition on its functionality. Analysis of functional gene annotations from the KEGG database revealed that the functional genes associated with heavy metal exposure and smoking groups were predominantly allocated to categories such as Membrane Transport, Carbohydrate Metabolism, Amino Acid Metabolism, Replication and Repair, Translation, Energy Metabolism, Xenobiotics Biodegradation and Metabolism, Lipid Metabolism, and Nucleotide Metabolism. This indicated that functional pathways such as Carbohydrate Metabolism, Translation, and Amino Acid Metabolism are oral microecological important functions (Wang et al., 2023). Research found the Nervous System and Biosynthesis of Other Secondary Metabolites were significantly enriched in the smoking group, while Xenobiotics Biodegradation and Metabolism, Amino Acid Metabolism, and Lipid Metabolism were significantly enriched in the heavy metal group. Co-exposure to heavy metals and cigarette smoking had a similar effect on function as heavy metal exposure alone. Heavy metals (HMs) could expedite the onset of oral diseases through diverse mechanisms, encompassing the disruption of cell membrane integrity, inhibition of enzyme activity, and induction of inflammation (Zhang Y. et al., 2023). Similarly, the presence of copper ions can inhibit adhesion of *Prevotella* to epithelial cells (Mihaila et al., 2019). In addition, HMs ions might play a role in cellular activities such as gene transcription, translation, and metabolism, accelerating the cell cycle of bacterial cells and contributing to the enhancement of bacterial adaptation to changes in the oral microenvironment induced by heavy metal ions. This, in turn, affected the stability of the oral microbiota, ultimately influencing the oral microbiota of individuals exposed to heavy metals. Thus, our study suggested that heavy metal and smoking exposures could induce dysbiosis in oral flora, thereby altering the function of bacterial communities. However, since PICRUSt2 predicted microbial function based only on 16S rRNA reads, the study was only a preliminary prediction of bacterial function, and further verification should be carried out in future studies using methods such as metagenomics to better understand the function of buccal mucosal bacteria from different populations.

The findings of this investigation revealed that heavy metals (Cd, Pb, Mo, Hg, Zn, Co) present in blood were associated with the proliferation of *Rhodococcus*, *Delftia*, *Porphyromonas*, *Granulicatella*, *Streptococcus*, *Neisseria*, *Gemella*, *Haemophilus*, *Peptostreptococcus*, with environmental factors eliciting alterations in the structural composition of bacterial communities. Consequently, heightened levels of heavy metals in the bloodstream might contribute to the onset of oral diseases by impacting the abundance and diversity of the oral microbiota. Environmental factors further disrupt the balance of the human oral microbiota, thereby compromising microbial interactions and predisposing individuals to oral diseases (Abdelhamid, 2016). Prolonged exposure to heavy metals such as nickel, cobalt, lead, and chromium had been reported to affect the expression of pro-inflammatory mediators, consequently precipitating the destruction of periodontal tissues (Khalid and Abdollahi, 2020). In a prior study, chronic lead exposure was associated with adverse impacts on oral health. Elevated levels of lead in the bloodstream were found to correlate with heightened expression of bacteria harboring *Streptococcus gordonii*, *Clostridium nucleatum*, and *Porphyromonas gingivalis*, which could lead to disease (Peyyala et al., 2018; Tort et al., 2018; Ayangbenro and Babalola, 2017). Even at low concentrations, salivary HMs can impair oral health and alter the bacterial community structure (Lu et al., 2014). Arsenic, a common HMs contaminant, had been reported to alter the intestinal flora of mice, resulting in a significant decrease in the abundance of Firmicutes (Li et al., 2019; Hsieh et al., 2018). While there remained a scarcity of studies substantiating the literature concerning the impacts of heavy metals (HMs) exposure on the composition of oral microbial communities, it was undeniable that heavy metal exposure alters the gut microbiota. Furthermore, the acknowledged homology between the oral and gut microbiota underscores the potential for heavy metal exposure to influence oral microbial communities. Additionally, contamination of soil with heavy metals inevitably led to alterations in soil microbial community structure, subsequently impacting the human body via the food chain (Li H. et al., 2023).

Network analysis could reveal complex interactions between different bacterial species in microbial ecology studies (Faust and Raes, 2012). It was found that the bacterial network of the heavy metal exposure group (M group) had fewer nodes and links than the bacterial networks of the other groups, suggesting that heavy metals lead to a simpler oral bacterial network and we found that the number of modules and nodes within each module in the network of the control group was higher than that of the other three groups, suggesting that the microbial network of the control group had higher complexity and ecological diversity, and that microbes interacted with each other in a more complex and tightly knit manner. Under the network construction conditions, most of the links were positive, and the node correlation coefficient cut-off value was >0.5, suggesting that the human oral microbiota might be predominantly co-operative or mutually beneficial to each other. It had been found that bacterial networks with lower graph density in megacities were more fragile than in non-megacities, and that megacity populations had more associations with oral-related diseases (Kim et al., 2018). Similarly, our results showed that the graph density of bacterial networks in the heavy metal and smoking co-exposure group was lower than that of bacterial networks in the other groups. Therefore, people in the heavy metal and smoking co-exposure group might be more susceptible to the risk of related diseases. The average degree value of the heavy metal exposure group was 3.111. The above results suggest that heavy metal exposure reduces the complexity of the oral mucosal bacterial network.

Based on Bayesian kernel machine regression (BKMR) modeling, we found an association between combined exposure to heavy metals and smoking and heavy metal exposure. Previous studies had shown that smoking and heavy metals affect the oral microbiome of populations. A large study of healthy people in the United States found that smoking affected the relative abundance of microbes in the phylum Actinobacteriota and Proteobacteria (Wu et al., 2016). Another study in a healthy population found that heavy metal exposure led to a reduction in the oral microbiome in the phylum Firmicutes, Proteobacteria, and Fusobacteriota (Al-Zyoud et al., 2020). This was consistent with the results of this study. These bacteria might be reduced by heavy metals and toxic substances in cigarettes, or indirectly by competition for colonization with enriched bacteria or co-aggregation with reduced bacteria. There were several potential mechanisms by which smoking and heavy metals may alter microbial ecology, including increasing the acidity of saliva, depleting oxygen, antibiotic effects, influencing bacterial adherence to mucosal surfaces, and impairing host immunity. Our analysis of inferred metagenomes revealed the combined heavy metal and smoking exposure group (MS) had a greater effect on the Cellular Processes, Environmental Information Processing, Metabolism, and Human Diseases than the heavy metal exposure group (M). Heavy metals and cigarette smoking were also known to have a significant impact on human immunity and thus on the host’s ability to resist pathogen colonization. The chemotactic mobility and phagocytosis of oral polymorphonuclear leukocytes were reduced in heavy metal-contaminated and cigarette smokers; since these cells were essential for host defence against pathogens, the harmful substances in heavy metals and smoke inevitably promote an oral ecosystem that was more conducive to pathogens, thereby increasing the risk for oral diseases.

In conclusion, based on Bayesian kernel machine regression modelling, we found that both exposure to heavy metal and smoking have an impact on oral microorganisms. However, our study indicated that heavy metals exposure exerted a more pronounced influence, with smoking playing a synergistic role.

## 5 Conclusion

We found that co-exposure to heavy metals and smoking altered the diversity of oral microflora more than heavy metals and smoking alone. Comparing the changes in oral bacterial community and functional pathways, there were significant differences in the oral microflora of different taxa, with each group having its own specific high abundance of bacteria. The effects of exposure to heavy metals were greater than those of smoking, and the effects of simultaneous exposure to heavy metals and smoking were slightly greater than exposure to heavy metals alone. Smoking plays a synergistic role in this study. Meanwhile, through the correlation analysis between microbiota and blood heavy metals, we found that the effect of Cd on microorganisms in this region was much greater than that of other heavy metals. This may be attributed to the severe pollution caused by environmental cadmium due to the long-term smelting of non-ferrous metals in the last century, despite the adoption of industrial measures. Despite measures such as industrial restructuring and ecological restoration, the environmental Cd pollution caused by past industrial production still cannot be recovered for a long time. This study will provide new ideas for oral health care and disease control measures for the population residing in the Baiyin area.

## Data Availability

The data presented in the study are deposited in the NCBI database repository, accession number PRJNA979792, https://www.ncbi.nlm.nih.gov/.

## Footnotes

^1^ https://gephi.org/
