# Supplementary material for: The impact of interactions between heavy metals and smoking exposures on the formation of oral microbial communities
Source: Front Microbiol. 2024 Dec 18;15:1502812. doi: 10.3389/fmicb.2024.1502812 (PMC11688307; doi:10.3389/fmicb.2024.1502812)
Supplement: Supplementary file 1 [file Table_1.docx]

**Supplementary Material for article:**

**The Impact of synergistic interactions between heavy metals and smoking exposures on the formation of oral microbial communities**

Qiwen Zheng ^1†^, Yonghua Zhang^2†^, Jia Li^1^, Shuwei Pei^1^, Jiangyun Liu^1^, Lu Feng^3^, Li Zhang^1^, Xingrong Liu^1^, Bin Luo^1^, Ye Ruan^1^, Weigang Hu^4^, Jingping Niu^1*^ and Tian Tian^1*^

^1^School of Public Health, Lanzhou University, Lanzhou, Gansu, 730000, The People's Republic of China.

^2^Child Health Department, Lanzhou Maternal and Child Health Care Hospital, Lanzhou, Gansu, 730000, The People's Republic of China.

^3^School of Stomatology, Lanzhou University, Lanzhou, Gansu, 730000, The People's Republic of China.

^4^State Key Laboratory of Herbage Improvement and Grassland Agro-ecosystems, College of Ecology, Lanzhou University, Lanzhou, 730000, The People's Republic of China.

^†^Co-first author, contributed equally to this work;

^*^Corresponding author: niujingp@lzu.edu.cn (J. Niu); tiant@lzu.edu.cn (T. Tian)

Supplementary Information Content:

Supplementary Figure (S1-S2)

Supplementary Table (S1-S6)


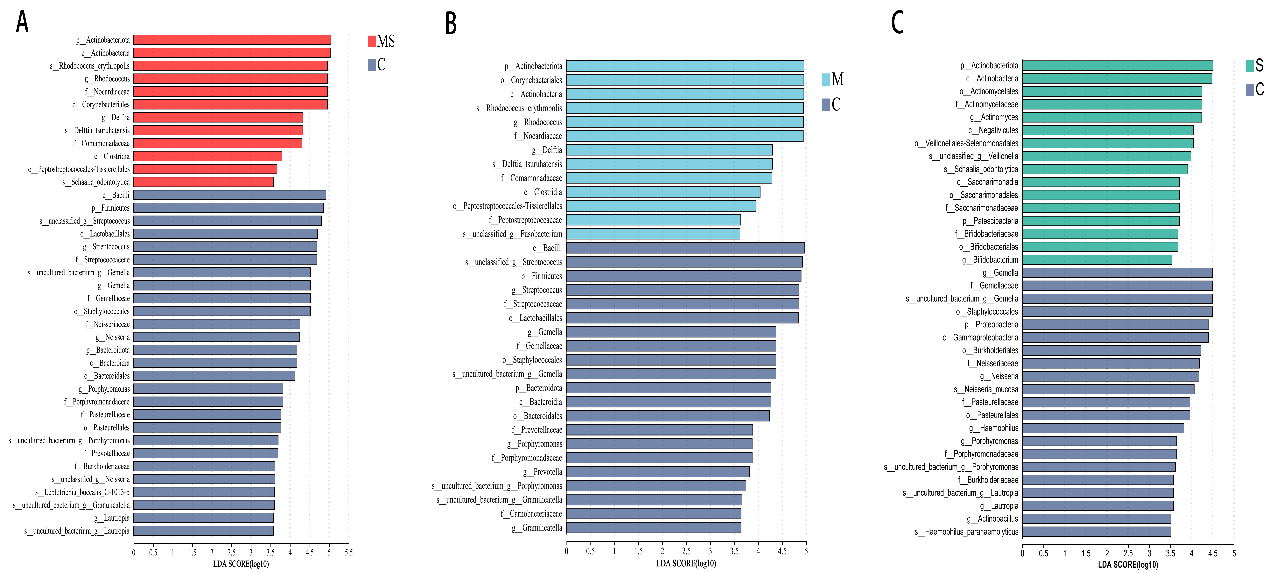
 **Fig. S1.** LEfSe analysis to select species that differed between groups. (A) Histogram of lefse difference species between MS and C groups. (B) Histogram of lefse difference species between M and C groups. (C) Histogram of lefse difference species between MS and C groups (MS = co-exposure to heavy metals and smoking, M = exposure to heavy metals, S = exposure to smoking, and C=control group).


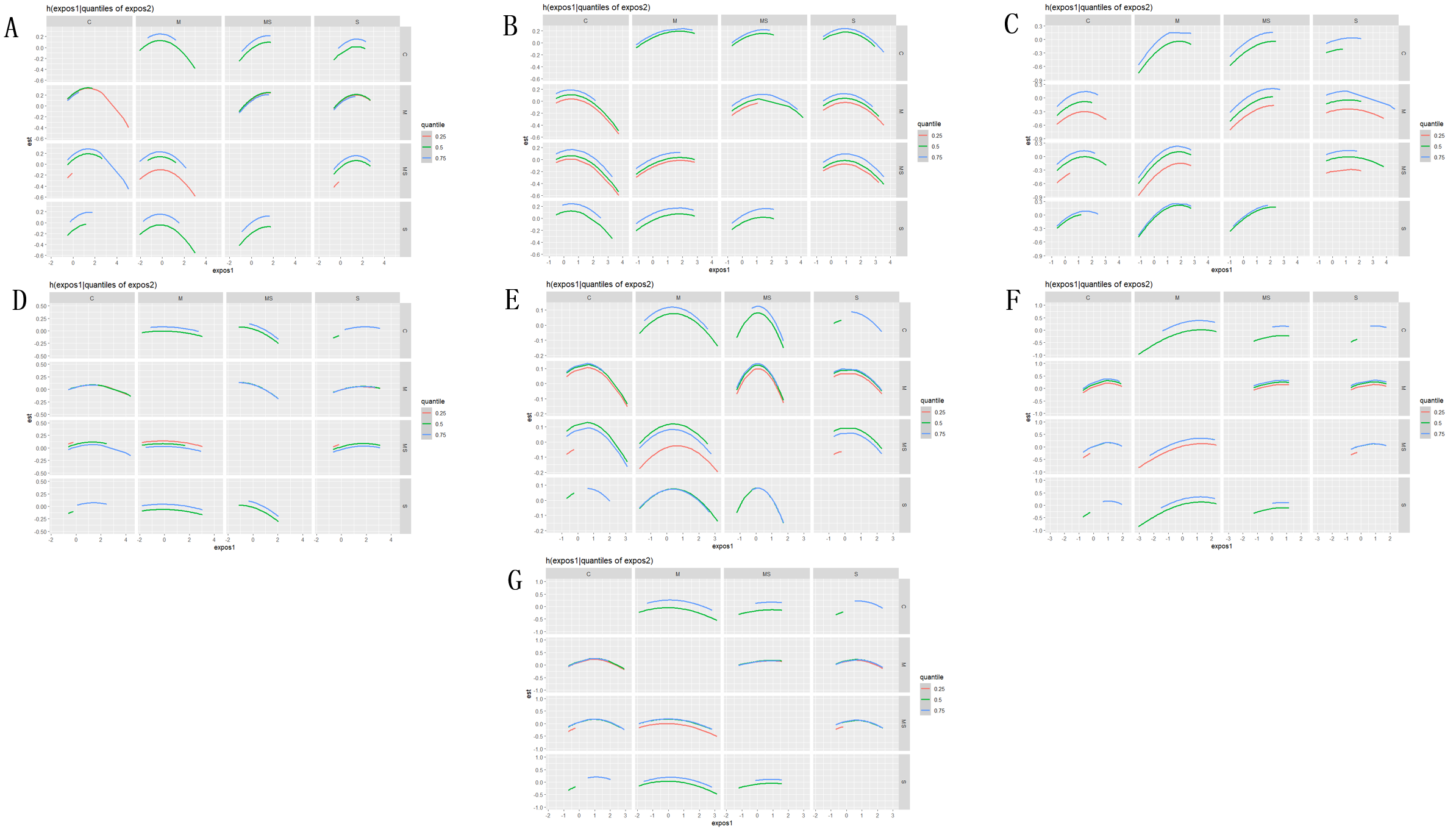
 **Fig. S2.**The interaction function among two exposure factor on bacteria phylum levels and KEGG Primary Pathways. The results were assessed by Bayesian Kernel Machine Regression (BKMR) models. The first three graphs show the effect on the phylum level of bacteria, these are the following Actinobacteriota (Fig. S2A), Patescibacteria ((Fig. S2B), and Proteobacteria ((Fig. S2C). The latter are based on the effects on the KEGG pathway, namely Cellular Processes ((Fig. S2D), Environmental Information Processing ((Fig. S2E), Metabolism ((Fig. S2F), and Human Diseases ((Fig. S2G). The results were assessed by Bayesian Kernel Machine Regression (BKMR) models.

**Table S1.** The concentration of metals in soils between contaminated and control areas (unit: mg/kg).

| Element | B (n = 6) | Y (n = 7) | *P*-value |
| --- | --- | --- | --- |
| Mo | 0.27 ± 0.06 | 0.09 ± 0.01 | 0.001* |
| Cd | 7.81 ± 5.21 | 0.11 ± 0.01 | 0.001* |
| Sb | 0.02 ± 0.00 | 0.00 ± 0.00 | 0.001* |
| Cu | 96.99 ± 25.45 | 18.00 ± 1.00 | 0.001* |
| Zn | 527.36 ± 313.25 | 47.63 ± 3.68 | 0.001* |
| Pb | 168.36 ± 49.94 | 12.98 ± 0.82 | 0.001* |
| Hg | 0.52 ± 0.15 | 0.02 ± 0.01 | 0.002* |
| Co | 8.29 ± 0.96 | 9.66 ± 0.64 | 0.181 |
| Ni | 31.31 ± 4.70 | 24.25 ± 1.54 | 0.234 |
| Mn | 514.00 ± 51.79 | 431.61 ± 33.06 | 0.534 |

Note: B, contaminated areas; Y, control areas. Data were shown as mean ± SE. **P* < 0.01.

**Table S2.** The concentration of metals in the blood of subjects living in both contaminated and control areas (unit: ng/ml).

| Element | B (n = 63) | Y (n = 16) | *P*-value |
| --- | --- | --- | --- |
| Mo | 2.34 ± 0.25 | 2.15 ± 0.53 | 0.506 |
| Cd | 8.62 ± 0.65 | 1.91 ± 0.43 | 0.000*** |
| Sb | 0.10 ± 0.01 | 0.10 ± 0.02 | 0.966 |
| Cu | 816.78 ± 14.06 | 776.46 ± 34.43 | 0.145 |
| Zn | 5831.07 ± 117.48 | 5238.74 ± 196.88 | 0.025* |
| Pb | 52.55 ± 2.67 | 18.42 ± 1.13 | 0.000*** |
| Hg | 0.56 ± 0.03 | 0.42 ± 0.04 | 0.015* |
| Co | 0.50 ± 0.07 | 0.37 ± 0.04 | 0.908 |
| Ni | 4.18 ± 0.77 | 4.89 ± 2.27 | 0.351 |
| Mn | 18.83 ± 1.33 | 15.62 ± 0.93 | 0.390 |

Note: B, contaminated areas; Y, control areas. Data were shown as mean ± SE. **P* < 0.05, ***P* < 0.01, ****P* < 0.001.

**Table S3.** Permutational multivariate analysis of variance (PERMANOVA) was used to compare oral bacterial community compositions and structures among different groups based on weighted and unweighted UniFrac distances (Differences shown in bold are statistically significant (*p* < 0.05)）

|  | Weighted | Unifrac | Unweighted | Unifrac |
| --- | --- | --- | --- | --- |
|  | R^2^ | *P* | R^2^ | *P* |
| Heavy metals | 0.147 | **0.001** | 0.054 | **0.001** |
| Smoking | 0.023 | **0.033** | 0.015 | **0.013** |
| indigestion | 0.004 | 0.793 | 0.008 | 0.382 |
| antibiotics | 0.005 | 0.633 | 0.007 | 0.736 |

**Table S4.** PERMANOVA analysis of intergroup differences in microbial communities (*P < 0.05), (***P* < 0.01), and (****P* < 0.001)

|  | Bray | Curtis |
| --- | --- | --- |
|  | R^2^ | *P* |
| C vs.S | 0.070 | **0.01*** |
| C vs.M | 0.167 | **0.001***** |
| C vs.MS | 0.205 | **0.001***** |

**Table S5.** Relative abundance of oral bacteria in different groups at the Phyla level (relative abundance <1% classified as others) (MS = co-exposure to heavy metals and smoking, M = exposure to heavy metals, S=exposure to smoking, and C = control group)

| Relative abundance | MS | M | S | C |
| --- | --- | --- | --- | --- |
| Firmicutes | 45.13% | 45.49% | 57.72% | 61.56% |
| Actinobacteriota | 35.13% | 29.78% | 19.08% | 13.11% |
| Proteobacteria | 11.07% | 14.59% | 7.86% | 12.59% |
| Fusobacteriota | 3.63% | 5.61% | 5.62% | 4.84% |
| Bacteroidota | 2.97% | 2.26% | 5.94% | 5.68% |
| Patescibacteria | 1.65% | 1.87% | 2.71% | 1.47% |
| others | 0.42% | 0.41% | 1.08% | 0.74% |

**Table S6**.Relative abundance of oral bacteria in different groups at the Genera level (relative abundance <1% classified as others) (MS = co-exposure to heavy metals and smoking, M = exposure to heavy metals, S = exposure to smoking, and C = control group)

| Relative abundance | MS | M | S | C |
| --- | --- | --- | --- | --- |
| Streptococcus | 29.92% | 26.64% | 38.96% | 41.66% |
| Rhodococcus | 22.01% | 19.87% | 5.30% | 3.55% |
| Gemella | 3.93% | 6.49% | 4.47% | 11.04% |
| Rothia | 6.54% | 3.13% | 4.32% | 5.15% |
| Actinomyces | 4.64% | 4.32% | 6.68% | 3.12% |
| Delftia | 5.54% | 4.91% | 1.63% | 1.27% |
| Neisseria | 2.14% | 3.15% | 2.25% | 5.15% |
| Haemophilus | 2.51% | 4.36% | 2.37% | 3.24% |
| Fusobacterium | 2.52% | 3.38% | 2.65% | 2.38% |
| Veillonella | 2.46% | 2.08% | 3.54% | 1.56% |
| Leptotrichia | 1.10% | 2.22% | 2.87% | 2.42% |
| Prevotella | 1.62% | 1.04% | 3.36% | 2.35% |
| Granulicatella | 1.76% | 1.46% | 2.42% | 2.42% |
| Porphyromonas | 0.91% | 0.76% | 1.35% | 2.13% |
| Peptostreptococcus | 0.96% | 1.62% | 1.26% | 0.86% |
| others | 11.43% | 14.56% | 16.58% | 11.71% |
